# The EU passes the AI Act and its implications for digital medicine are unclear

**DOI:** 10.1038/s41746-024-01116-6

**Published:** 2024-05-22

**Authors:** Stephen Gilbert

**Affiliations:** Else Kröner Fresenius Center for Digital Health, TUD Dresden University of Technology, Dresden, Germany

**Keywords:** Health policy, Health services, Medical ethics

## Abstract

On 13 March 2024, the much-anticipated AI Act was passed by the EU parliament and will soon be adopted as EU law. It will apply new requirements for developers and deployers of AI-enabled digital health tools (DHTs), including for a defined class of high-risk AI systems and for general-purpose AI. Although the text of the law is available, complete in all but the final checks of legal wording much is still not known about how the AI Act will affect the digital health landscape in the EU and beyond. The wording of many aspects of the Act is ambiguous, and often high-level objectives are stated, with the detail to come later in associated guidance, standards, and member state law and policy. It is also uncertain how the Act will intersect with pre-existing sector-specific legislation for medical AI. There are future steps in the legislative process that can clarify ambiguity, including standards, guidelines, and implementing laws, and the author remains optimistic the EU will get the implementation right.

On 13 March 2024, the much-anticipated AI Act was passed by the EU parliament to become law. The new law will soon, after checks of final legal wording, pass into force, and it will apply to the developers and deployers of general-purpose AI systems in 12 months, to AI systems including AI-enabled digital health tools (DHTs) in 24 months, and to high-risk AI systems, including DHTs in 36 months^1,2^. The Act brings with it many new responsibilities for developers, deployers (Table 1), notified bodies, and regulators for the newly established EU AI Office^1,2^ and for the European Commission. As with other EU legislation, if will even have an impact on DHTs developed, placed on the market, and put into service outside the EU^1^. The scope excludes “AI systems and models specifically developed and put into service for the sole purpose of scientific research and development”^1^.

**Table 1:** Summary of new responsibilities of developers and deployers brought in by the AI Act

**#**	**Nature of principal new responsibilities of developers, deployers of AI-enabled software products and platforms even “certain AI systems … when they are neither placed on the market, nor put into service, nor used in the Union.”**^1^
1	Mandatory requirements to observe the principles of: (i) human agency and oversight; (ii) technical robustness and safety; (iii) privacy and data governance; (iv) transparency; (v) promoting equal access, gender equality and cultural diversity, while avoiding discriminatory impacts and unfair biases; and (vi) promoting social and environmental well-being.
2	AI Systems must meet requirements for transparency, ‘*meaning that the that AI systems are developed and used in a way that allows appropriate traceability and explainability, while making humans aware that they communicate or interact with an AI system*’^1^.
3	There is an explicit requirement that not only the risks to health and safety must be considered throughout the entire product life cycle, but also the risks to “*fundamental rights*” It is not entirely clear if the latter refers only to the rights of the affected person, or also to the staff using the AI system. *Although this is a new explicit requirement, most healthcare AI system developers will have considered this in their compliance to medical product legislation and in order to comply with GPDR*^10^ .
4	‘*The requirement for the datasets to be, to the best extent possible, complete and free of errors*’^1^
5	Oversight of systems is required: ‘*where appropriate, such measures should guarantee that the system is subject to in-built operational constraints that cannot be overridden by the system itself and is responsive to the human operator*.’^1^

Some of these responsibilities overlap existing responsibilities under vertical product legislation. The responsibilities described here are taken directly from the Act^1^, which also contains many other new responsibilities for other stakeholders which are not described here.

Although the initial definition proposed for AI in the act was wide, encompassing many simple software functions not based on learning from data^3^, the final definition settled on is of systems with “*capability … to derive models and/or algorithms from inputs/data*”^1^. The AI Act is a so-called ‘horizontal legislation’, which is known as such as it applies across the wide span of all sectors of human industrial and economic activity. Although the law is agreed, much remains unknown of its final interpretation and implications, as ways must be found to apply it alongside the pre-existing single-sector legislation (i.e., the ‘vertical legislation’), which for healthcare domain are principally the Medical Device Regulation (MDR)^4^ and In-Vitro Diagnostic Device Regulation^5^. The MDR and IVDR will continue to apply healthcare AI alongside the AI Act^6^. The true implications of the AI Act will only be discovered in the laboratory of the real world in the months, years, and decades to come. This article gives a high-level summary of the history, aims, controversies, and most challenging heat points that will likely define the actual impact of the law.

The AI Act, which was published in draft form on 21 April 2021^3^, sets out a framework for the governance of the application of the development of AI in the EU. It aims to set out: (a) uniform internal market rules “for the development, placing on the market, putting into service and the use of artificial intelligence” in a manner that (b) promotes ‘*human centric and trustworthy artificial intelligence*’ and (c) ‘*ensuring a high level of protection of health, safety, fundamental rights*’^1,2^. Although challenging, it is within the realm of the possible for a law to achieve all of (a) through (c). The great challenge and the ‘trillion-euro question’ (upon which the future of the EU as a power that can develop novel AI technologies), is how the law achieves goals (a) through (c) while also delivering (d), which is the simultaneous promotion of and support for innovation. This is not an impossible task but certainly one in which previous EU laws relating to the medical sector and digital medicine have fallen far short^6^.

There has been much frustration within the EU healthcare AI sector and industry on the entire concept of a horizontal ‘AI Act’, with many stakeholders expressing the view that the existing ‘vertical’ legislation (the MDR^4^ and IVDR^5^) could be adapted for the AI age. These vertical laws regulate AI-enabled software as a ‘medical device’ where the product is intended by the developer to enable decision-supporting outputs, including in the areas of diagnosis, prevention, monitoring, prediction, prognosis, treatment, or alleviation of disease, and in medically related lifestyle adaptation^4,5^.

## What does this new law mean for Digital Medicine?

The AI Act is written in a highly readable style, and in my view, most of the individual paragraphs, articles, and annexes (but likely not their implications) are readily understandable to the layperson. As a horizontal legislation on one of the most pervasive sets of technologies ever conceived by mankind, the AI Act is by necessity a long document and has many considerations for specific AI applications. This article summarizes the considerations principally applicable to digital medicine only. The AI Act has 80 pages of preliminaries, then 85 detailed Articles set out in 140 pages, followed by 14 Annexes in 30 pages. Reading this induces a feeling of nausea and information overload, but, in my view, there is barely a paragraph that does not address a critical challenge that the AI age brings with it. Pain points in its negotiation (Table 2) included the definition of AI and how the oversight of AI systems will be achieved (Article 14)^1^. There was much discussion, and some slowing of the development of the Act in November 2023 due to intensive negotiations between EU member states on the treatment of generative AI and foundations models under the Act^7^, an understandable point of controversy due to the rapid development of these technologies in 2022 and 2023, and questions about how their innovative nature should be regulated.

**Table 2:** Summary of pain points in the negotiation of the AI Act relevant to digital medicine and how they were resolved

#	The context of the negotiation problem	The proposed remedy or compromise
1	**The definition of AI** The definition of AI (or types of AI) in scope, which initially used a definition that included many simple software decision approaches, used in much medical device software, and not conventionally described as AI.	A more conventional definition of AI was adopted that specifies that ‘AI systems’: (1) derive models and/or algorithms from inputs/data. (2) use techniques that “*enable inference including machine learning approaches that learn from data how to achieve certain objectives” or “logic- and knowledge-based approaches that infer from encoded knowledge or symbolic representation of the task to be solved*”. (3) “*have the capacity … to infer [that] goes beyond basic data processing’*
2	**Foundation and general models** The technologies and importance of foundation models and ‘General purpose AI’ (GPAI) models advanced greatly in the period after publication of the draft AI Act^3^, and was a controversial area requiring much negotiation^7^	There are special requirements for the EU AI Office and authorities for the market surveillance of GPAI when these can be used for (even one) high risk purpose
3	**What is high risk?** The definition of high-risk AI (in Annex III of the Act) applies to any digital healthcare AI system used in relation to diagnosis or therapy, even if these are supportive/informational suggestions of options to consider. This definition would include many AI approaches that would in the reality of their use, be highly subject to human revision, adaption and improvement.	Criteria were added that exclude certain AI use cases from the high-risk category, including those that are intended: (1) to perform a narrow procedural task: such as an AI system that transforms unstructured data into structured data, that classify incoming documents into categories, or are used to detect duplicates among a large number of documents. (2) to improve the result of a previously completed human activity such that it only provides an additional layer to a human activity with consequently lowered risk. (3) to detect decision-making patterns or deviations from prior decision-making patterns. Here the risk would be lowered because the use of the AI system follows a previously completed human assessment which it is not meant to replace “or influence, without proper human review.” (4) to perform a task that is only preparatory to an assessment

## Known and unknown unknowns

Even after the law was agreed in the legislative process and the near-complete final text was published, there remain many issues that are uncertain and many areas of potential conflict with other laws. One of the most important known unknowns is the final form and influence of ‘AI regulatory sandboxes’^1^. These are defined as ‘*a concrete and controlled framework set up by a competent authority which offers providers or prospective providers of AI systems the possibility to develop, train, validate and test, where appropriate in real-world conditions, an innovative AI system, pursuant to a sandbox plan for a limited time under regulatory supervision’*^1^. Although these are described in many sections of the AI Act, and it is required that national authorities establish at least one of these, the detail on how these will be implemented is yet to be determined, and much has been left to individual member states.

Any new law also brings with it ‘unknown unknowns’—areas that are not anticipated, many of which were not reasonably foreseeable by the legislator and need to be addressed in implementing legislation, standards, or guidance. These are also not foreseeable in this article, but we can anticipate that, due to the horizontal nature, wide scope, and rapidly changing nature of healthcare AI, there will be many problems created by ambiguities and uncertain intersections with existing laws. Here, flexibility and intelligence of the responsible bodies, including the Commission, the EU AI Office^1,2^, the committees responsible for creating guidelines for medical device software under the MDR, and even of the courts will be essential to meeting the stated aim of the AI Act, to promote rather than to decimate AI sector entrepreneurial activity and innovation.

## Summary

The view of the author is that this horizontal AI Act was essential, as AI is itself transversal and horizontal in its reach, pervasiveness, and inherent intersectoral connected implications—examples of this (that I have described elsewhere) are the sector-spanning implication of AI in medical devices linking to increasingly adaptive (at the patient bedside) pharmaceuticals^8^ and the increasingly real-time intersection between medical AI and wellness AI, via sensors, wearables and smartphones and their apps^9^. As described above, many stakeholders view the AI Act as an unwelcome and unnecessary imposition. Which side of this debate was right is now a question confined to the dustbin of history, as the AI Act has passed into law, and all in the sector, even outside the EU, must now grapple with its implementation and impact. I believe that the law, as written, could be well implemented and could achieve its seemingly paradoxical aims of promoting human-centric trustworthy AI, protecting health and fundamental rights, while simultaneously promoting innovation, if (and only if) intelligence and flexibility and fleet-of-foot in reaction to emergent problems are demonstrated by Act’s governance structures. Whether that can be achieved is an open and critical question, and the European Commission must ensure that there is no pause between the ‘victory’ of passing the act and the construction of this future state that enables an ethical, equitable, and (as important as the other two) entrepreneurial AI-enabled digital medicine future for the EU.
